# How few and far between? Examining the effects of probe rate on self-reported mind wandering

**DOI:** 10.3389/fpsyg.2013.00430

**Published:** 2013-07-17

**Authors:** Paul Seli, Jonathan S. A. Carriere, Merrick Levene, Daniel Smilek

**Affiliations:** Department of Psychology, University of WaterlooWaterloo, ON, Canada

**Keywords:** mind wandering, thought probes, thought sampling, metronome response task, MRT

## Abstract

We examined whether the temporal rate at which thought probes are presented affects the likelihood that people will report periods of mind wandering. To evaluate this possibility, we had participants complete a sustained-attention task (the Metronome Response Task; MRT) during which we intermittently presented thought probes. Critically, we varied the average time between probes (i.e., probe rate) across participants, allowing us to examine the relation between probe rate and mind-wandering rate. We observed a positive relation between these variables, indicating that people are more likely to report mind wandering as the time between probes increases. We discuss the methodological implications of this finding in the context of the mind-wandering literature, and suggest that researchers include a range of probe rates in future work to provide more insight into this methodological issue.

## Examining the effects of probe rate on self-reported mind wandering

Mind wandering is, by definition, a subjective experience. Given its subjective nature, it is common practice for researchers who study mind wandering to identify its occurrence by using “thought-sampling” methods that require participants to introspect and report when they find themselves thinking about task-unrelated concerns (see Smallwood and Schooler, 2006). One of the more popular thought-sampling methods used in studies of mind wandering is the probe-caught method (Giambra, 1995; Schooler et al., 2005; etc., see Smallwood and Schooler, 2006 for a review), which involves interrupting a given task with randomly or strategically presented probes that explicitly ask participants to indicate whether they were, just prior to the onset of the probes, mind wandering or focused on the occurrent task. To date, numerous studies have shown robust associations between probe-caught measures of mind wandering and performance on various different tasks (e.g., Smallwood et al., 2004, 2008; Christoff et al., 2009; Seli et al., 2013a), validating the probe-caught method as an index of mind wandering.

One consideration that inevitably arises when designing an experiment using the probe-caught method is the rate at which the thought probes should be presented. On the one hand, if there is too much time between each of the probes, then relatively few probes can be presented, and as a consequence, thoughts might not be sampled at a high enough frequency to provide a reliable index of mind wandering. On the other hand, we have found ourselves reasoning that placing thought probes too close together in time might be problematic because this might not provide enough time for the mind to wander away from the task at hand. We find this latter consideration to be interesting because it reveals a critical implicit assumption: Namely, that the temporal rate at which thought probes are presented might affect the likelihood that participants will report being in a state of mind wandering. If this assumption is in fact valid, then it would have important methodological implications for mind-wandering researchers because it would indicate that comparisons of mind-wandering rates across studies ought to take probe rate into account.

In the present experiment, we explored the effect of probe rate (i.e., the temporal intervals between probes) on mind-wandering rate by varying average probe rates across participants while they completed a sustained-attention task (the Metronome Response Task; MRT; Seli et al., 2013a). For the MRT, participants are required to attentively keep time with a metronome by pressing a button on a keyboard or mouse in synchrony with the onset of each metronome tone; variability in MRT responses has been associated with mind wandering (Seli et al., 2013a). Here, for the first time, we administered the MRT online and so we initially sought to determine whether the online results were similar to those collected in the laboratory. Given our aforementioned assumption that placing probes too close in time might not allow sufficient time for one's mind to wander away from the task at hand, we expect to observe a decline in probe-caught mind wandering as the time-between-probes decreases. It is, however, possible that we will instead observe an increase in probe-caught mind wandering with decreases in time-between-probes because the relatively frequent probing associated with short periods of time-between-probes might be perceived as very disruptive, thereby preventing people from focusing on the primary task.

## Method

### Participants

Participants were 126 individuals who completed a Human Intelligence Task (HIT) posted on the Amazon Mechanical Turk (www.mturk.com). Participants were paid $.75 for completing the HIT, which lasted ~20 min and consisted of brief demographic and mind-wandering questionnaires followed by the sustained-attention task. According to the ethical conduct guidelines for this study, participants were allowed to skip or discontinue performance of the attention task if they wished, and 22 participants did so. In at least some cases this was likely due to technical problems playing the metronome tone required for the sustained-attention task, though the reason for skipping the task was not logged. Of the 104 participants who were able to complete the full study, 101 were from the United States of America, 62 were female, and the mean age was 41.7 (*SD* = 13.7; Range = 19–68).

### Materials

#### Questionnaires

Demographic information was collected from each participant, including age, gender, occupation and country. We also assessed trait mind wandering via the brief Mind Wandering: Deliberate (MW-D) and Mind Wandering: Spontaneous (MW-S) scales (Carriere et al., 2013). These scales include items such as “I allow my thoughts to wander on purpose” (deliberate), and “It feels like I don't have control over when my mind wanders” (spontaneous), and participants respond using a seven point Likert-type scale.

#### Metronome response task (MRT)

The MRT (Seli et al., 2013a) is a sustained-attention task in which participants must attentively monitor the passage of time in order to provide a key-press response in synchrony with a periodic metronome tone. If one's attention fails at any time, then the estimation of when the tone will occur will be affected and the timing of one's responses becomes more variable. For this study, the MRT was run within the participant's web browser, using a mix of HTML 5, CSS, and JavaScript programming. In this case the timing accuracy of the metronome tone and participant responses are entirely under the control of the browser, and millisecond accuracy is unlikely to be obtained. Nonetheless, in pilot testing we observed no noticeable irregularity in the presentation rate of the metronome tones across a variety of systems and browsers. Participants were first required to test playback of the metronome tone, and were allowed to set their speaker volume to a subjectively comfortable level. If participants had any difficulty playing the test tone they necessarily skipped the task and were immediately taken to the feedback screen for the study. Participants who indicated success playing the test tone were instructed that they would be hearing the same metronome tone at a regular interval for ~15 min, and were to press the spacebar on their keyboard in time with the presentation of the tone. The MRT began with 18 practice trials in which the metronome tone was presented once every 1300 ms. Following the practice trials, participants received feedback on how accurately they had been responding in time with the tone. Participants typically responding more than 150 ms before or after the tone for the second half of these trials were advised to either wait longer or press sooner, as appropriate. Before beginning the remainder of the task, participants were further instructed that the metronome tones would occasionally stop, and at those moments they would be asked to report whether they were mind wandering. Additionally, participants were instructed to report that they had been mind wandering only if they were thinking about something that was *unrelated to the task*, such as what to eat for dinner or bills that need to be paid. After reading this additional instruction, participants completed a further 600 trials of the MRT.

Mind-wandering probes were distributed pseudo-randomly throughout the 600 trials of the MRT, as they would normally be for the MRT, with the only restriction being that they had to be at least 8 trials (10.4 s) apart. We were interested in examining the effects of probe rate on mind wandering, so for this version of the MRT the number of probes presented was varied between 5 and 25 across participants—this ensured participants received probes across a wide range of time intervals. Upon loading the MRT, the number of probes to be presented was checked out from a fully counterbalanced database and if the MRT was then skipped that value was checked back in, ensuring roughly equal representation across the full range. As there is a direct relation between task duration, the number of probes presented, and their randomly selected rate of presentation, for this study the maximum amount of time a participant went without receiving a probe was 4.6 min, while the mean was 50.6 s (*SD* = 24.2). Participants were not aware of the number of probes they would receive, nor were they aware of the rates at which they would be presented.

### Procedure

The study was completed in a new browser window, separate from the Mechanical Turk. Demographic information was collected first, followed by, in random order across participants, the two mind wandering questionnaires. The MRT was always presented after the questionnaires. At both the beginning and end of the study participants were provided a random, numeric completion code to be entered into the Mechanical Turk in order to receive payment for the HIT. Providing the code at the beginning of the study ensured that all participants could withdraw at any time by simply closing their browser window, without penalty.

### MRT measures

For the present study, the primary measure of interest obtained from the MRT is the Proportion of Mind Wandering (i.e., the number of probes to which a participant reported mind wandering, divided by the total number of probes presented). To ensure the MRT was generally being performed correctly, and that the participants' self-reported mind wandering was indeed a departure from performing the task at hand, we also calculated several task-related response measures. Rhythmic Response Times (RRTs) were first calculated as the difference of the time of the key press and the onset of the metronome; the Mean RRT therefore indicates whether, on average, participants' responses precede or succeed the metronome tone. As variability in response time is the main measure of attention yielded by the MRT, aside from probe-caught mind wandering, three measures of variance of the RRTs were also calculated. Our first measure of variability, Mean RRT Variability, was computed using a moving window of the current and preceding four trials across all trials throughout the task except the first five trials and five trials after each probe (see Seli et al., 2013b)^1^. Our second measure of variability was for the five trials preceding subjective reports of on-task performance (On-task RRT Variability). Finally, our third measure of variability involved the five trials preceding reports of mind wandering (Mind Wandering RRT Variability)^2^. Variance data are often highly positively skewed, so we adjusted each variance measure using a natural logarithm transform. In summary, for each participant, we calculated the variance in RRTs for a five trial moving window, throughout the task, calculated the mean of these windows, and then identified the last windows immediately prior to each probe in order to identify the degree of variability associated with on-task and mind wandering behavior and calculated the means of these as well.

## Results

Three participants had omission rates (i.e., missed responses) above the standard cut-off of 10% for the MRT (Seli et al., 2013a), and were removed from all analyses. For the remaining 101 participants, the means and standard deviations for each MRT and questionnaire measure are shown in Table 1. The MRT measures show good agreement with those previously obtained in the lab (Seli et al., 2013a,b), although we observed a slight elevation in the On-task RRT Variability measure, and a positive, rather than negative, Mean RRT. These are likely artifacts of running the MRT within a web browser, and losing the ability to run the program with millisecond-accurate precision. The most probable scenario is that the tone itself was being presented later than we intended and since we did not have the ability to measure the time at which the tone was actually played, we subtracted too small of a value when calculating the mean RRTs. Nevertheless, as it seemed to be a consistent delay, this should have had little, if any, effect on our ability to measure response variability. Importantly, the typical MRT finding of increased variability prior to self-reported mind wandering, compared with variability prior to reports of being on-task, was replicated. A two-tailed paired-samples *t*-test revealed a significant difference between these measures, *t*_(88)_ = 2.09, *d* = 0.23, *p* = 0.039, with mind wandering being associated with significantly greater variability, suggesting the participants in this online sample completed the task similarly to those in previous in-lab studies. In addition to the MRT measures, the mean responses on the mind wandering questionnaires also both showed good agreement with previous samples (Carriere et al., 2013).

**Table 1:** **Means (SD) for all MRT measures, and for trait mind wandering questionnaires (deliberate and spontaneous) (*N* = 101)**.

**Measure**	***N***	**Mean (*SD*)**
Mean RRT	101	50.9 (98.98)
Mean RRT variability	101	8.24 (0.66)
On-task RRT variability	94	8.09 (1.10)
Mind-wandering RRT variability	95	8.34 (0.91)
Mind Wandering: Deliberate	101	4.6 (1.54)
Mind Wandering: Spontaneous	101	4.0 (1.51)

A positive Mean RRT indicates responding after the metronome, measured in milliseconds. RRT Variabilities received a natural logarithm transformation to ensure a normal distribution. Not all participants reported at least one instance of mind wandering or being on task. In previous in-lab research (Seli et al., 2013a Sample 1) On-task RRT Variability was 8.00 (SD = 1.33), and Mind-wandering RRT variability was: Tuned out = 8.24 (SD = 1.29), Zoned out = 8.51 (SD = 1.37).

Having shown that the MRT performed well in an online sample, we next examined the relations among the key measures. Pearson product-moment correlations of the MRT and questionnaire measures are shown in Table 2. Here we observe a number of expected findings: namely, significant positive relations of self-reported (i.e., probe caught) Proportion of Mind Wandering with RRT Variability, MW-D, and MW-S, as well as a significant positive relation of MW-D and MW-S. Additionally, we observed a positive relation of RRT variability and MW-S, but not MW-D, suggesting that one's increased response variability due to mind wandering is a result of a tendency to engage in spontaneous rather than deliberate mind wandering. This makes good sense, as deliberate mind wandering may be a strategic form of mind wandering that one undertakes only when it is unlikely to interfere with primary task performance.

**Table 2:** **Pearson product-moment correlations of Mean MRT Variability, Proportion of Mind Wandering, Mean Time Between Probes, and trait mind wandering questionnaires (deliberate and spontaneous) (*N* = 101)**.

**Measure**	**Proportion of mind wandering**	**Time between probes**	**MW-D**	**MW-S**
Mean RRT variability	0.26^**^	−0.04	0.07	0.23^*^
Proportion of mind wandering		−0.26^**^	0.40^**^	0.49^**^
Mean Time Between Probes			0.10	0.17^3^
Mind Wandering: Deliberate				0.55^**^
Mind Wandering: Spontaneous				

* p < 0.05,

** p < 0.01.

**Figure 1:**
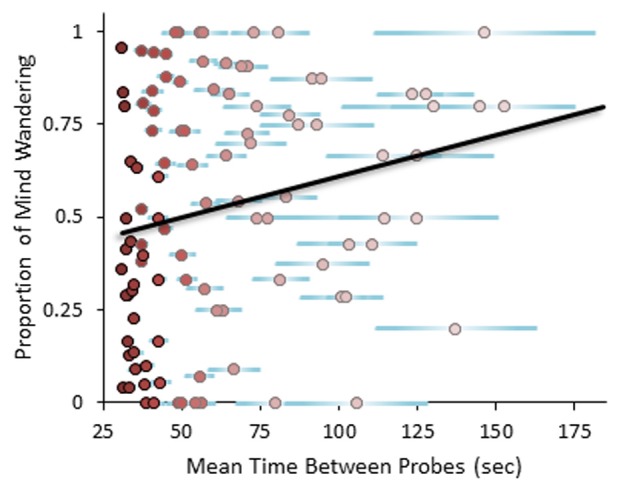
**Scatterplot showing linear relation of Mean Time Between Probes with proportion of self-reported mind wandering.** Each point represents one participant, while the point color indicates the number of probes provided (darkest = 25, lightest = 5). Horizontal error bars show one standard error of the Mean Time Between Probes.

For the purposes of the present study, the most important finding shown in Table 2 is the significant positive relation of Time Between Probes and self-reported Proportion of Mind Wandering during the MRT. The scatterplot shown in Figure 1 illustrates this effect, which indicates that if participants are probed more frequently, at around 30 s on average, we would expect reports of mind wandering about 46% of the time. If, however, participants are probed less frequently, at around 3 min on average, we would expect reports of mind wandering about 79% of the time. These findings are, of course, likely to be specific to performing the MRT, but it is interesting that more frequent probing results in less mind wandering, and to a similar extent as in other attention-demanding tasks (e.g., silent reading: Smilek et al., 2010; Uzzaman and Joordens, 2011). At longer durations without probes, however, the amount of reported mind wandering becomes incredibly high, indicating that participants may be biased to report more mind wandering in these cases—or, alternatively, that given a sufficiently boring task with few interruptions, participants are likely to disconnect attention from the task and spend the vast majority of their time mind wandering.

## Discussion

In the present experiment, we sought to determine whether the amount of time between thought probes affects probe-caught rates of mind wandering. To explore this possibility, we had participants complete a sustained-attention task (the MRT) during which we intermittently presented probes to sample for periods of mind wandering. Importantly, we varied the Mean Time Between Probes across participants to allow us to determine the effect, if any, of probe spacing (i.e., probe rate) on probe-caught mind-wandering rates. Given that this was the first time the MRT was administered online, we initially wanted to verify the online results replicated those previously found in the laboratory (i.e., Seli et al., 2013a). Consistent with previous (in-lab) results, we found greater response variance accompanying self-reported periods of mind wandering relative to on-task periods, demonstrating that MRT response variance can be used to detect mind wandering within an online sample. The present findings were also consistent with previous studies showing ~50% mind wandering at a probe rate of roughly one per minute (e.g., Smilek et al., 2010; Uzzaman and Joordens, 2011; Seli et al., 2013a); here we observed similar rates of mind wandering when probes were presented at the rate of one per minute. Finally, we observed that the mean values from the mind-wandering questionnaires (i.e., the MW-S and MW-D) were consistent with previous work (Carriere et al., 2013). Having established these consistencies among our online study and previous online and in-lab studies, we next examined the relation between the proportion of probe-caught mind wandering and Mean Time Between Probes. This analysis revealed a positive correlation, indicating that probe-caught mind wandering declines with decreases in the Mean Time Between Probes. Thus, as hypothesized, the amount of time between thought probes does in fact influence probe-caught reports of mind wandering, with less time between probes leading to fewer reports of mind wandering.

One important concern raised by the present results is whether different probe rates actually affect the experience of mind wandering (i.e., its incidence) or instead alter the likelihood of reporting mind wandering (i.e., produce a response bias). One aspect of the results that might shed light on this is the lack of a significant relation between Mean Time Between Probes and response variance in the MRT (Mean RRT Variability). This result is important because MRT variance has been shown to reliably index mind wandering, both in the present experiment and in previous studies (Seli et al., 2013a,b); thus, on the basis of these results, one would expect increases in Time Between Probes—which are ostensibly associated with increases in mind wandering—to also be associated with increases in MRT variance. As these variables were not related, it seems that varying the Time Between Probes might alter one's likelihood of reporting mind wandering without affecting the actual incidence of mind wandering itself. Of course, future work will be needed to determine whether this is indeed the case, as this outcome could also occur if MRT variance and self-reported mind wandering measure somewhat different aspects of mind wandering behavior and only one of those aspects is affected by the rate of probing (e.g., one engages in deliberate mind wandering to occupy spare cognitive capacity and fend off boredom, without impacting task performance). Assuming this is not the case, however, the results of the present work would have to be seen as favoring the response-bias explanation.

The present findings are of practical importance because, given the wide range of mind-wandering rates observed across our range of probe rates, they suggest that when comparing mind-wandering rates across experiments, researchers will have to take into account the different probe rates used. For example, Uzzaman and Joordens (2011) report a higher rate of mind wandering than Smilek et al. (2010), but their probes were also, on average, farther apart; thus, the higher rate of mind wandering they reported is predicted by the present findings. On a similar note, the results of the present work suggest that studies reporting mind-wandering incidence rates might need to be reevaluated, given that the reported mind-wandering rates may not be reflective of the objective rates, and might very well be markedly different if different probe rates were used. One possible way for future research to deal with these issues is to employ a range of thought-probe rates; this would provide important insights into the methodological aspects of mind-wandering research, and would perhaps produce a more representative measure of mind-wandering incidence. With respect to the concern about different probe rates producing different response biases, it will be important for future research to develop new techniques to separate response bias from actual mind wandering incidence.

### Conflict of interest statement

The authors declare that the research was conducted in the absence of any commercial or financial relationships that could be construed as a potential conflict of interest.

## Footnotes

^1^We used the moving-window analysis because it retained the temporal component of the variance across the task, while also limiting the influence of outlier responses on overall variability.

^2^When examining response variability prior to self-reports of both on-task performance and mind wandering, we used a five-trial window because: (1) we wanted to have a sufficient number of trials with which to compute variance, and (2) we did not want to sample data from trials that preceded the probes by too much time, because then we would run the risk of sampling behavior that was not proximal (and relevant) to the current thought probe.

^3^This relation is nearly significant, *p* = 0.08, but can only have occurred by chance as the number of probes presented was independent of MW-S scores. A first-order partial correlation of Prop. Mind Wandering with Time Between Probes controlling for MW-S was found to be statistically significant, *r*(100) = 0.21, *p* = 0.03.
